# Carious Exposure versus Mechanical Exposure for MTA Pulpotomy in Primary Teeth

**DOI:** 10.1155/2016/2753429

**Published:** 2016-11-22

**Authors:** Burcu Nihan Çelik, Şaziye Sarı

**Affiliations:** Department of Pedodontics, Faculty of Dentistry, Ankara University, Ankara, Turkey

## Abstract

*Introduction*. The etiology of exposure determines pulpal response, making it crucial to distinguish between mechanical and carious exposure. This study clinically and radiographically evaluated the success of MTA pulpotomies conducted to treat carious and mechanical pulp exposure.* Materials and Methods*. This study was conducted with 50 mandibular primary molar teeth. Teeth were divided into 2 groups according to status of the exposure site, with teeth surrounded by carious dentin placed in a carious exposure group and those surrounded by sound dentin in a mechanical exposure group. MTA pulpotomies were performed for both groups. Treatment was followed up clinically and radiographically for 18 months.* Results*. Clinical and radiographic success rates at 18 months were 100% for both groups. Success rates did not vary significantly between the groups (*p* = 1.000). Pulp canal obliteration was only seen in the carious exposure group, observed in 2 teeth (8.3%).* Conclusion*. The long term success rates achieved in this study indicate that MTA can be used as a vital pulpotomy material for the long term success in primary teeth with either mechanical or carious exposure. The findings of the present study highlight the fact that treatment prognosis is dependent upon diagnosis and selection of the appropriate materials for treatment.

## 1. Introduction

Accurate diagnosis of pulp inflammatory status is the key factor for predicting healing capacity and thus prognosis in vital pulpotomy treatment in primary teeth [1, 2]. The characteristics of the dentin surrounding the exposure site which can be defined as “nature of pulp exposure” may be critical for healing and repair capacity of the exposed pulp and it has been asserted that the size and nature of pulpal exposure should be evaluated as an integral component of pulpal diagnosis and treatment planning [3–9].

Indications for vital pulp therapies have been reported as “carious, mechanical, and traumatic pulp exposures” in clinical guidelines and reviews [10–12]. Additionally, nature of pulp exposure has been specified as “a carious or mechanical exposure of vital coronal pulp tissue” and detailed as “pulps of primary teeth are exposed during caries removal, or even when they are exposed iatrogenically or by trauma” [12].

The only detailed definition which belongs to Starkey [13] reported that exposure type can be determined when the last bit of caries is removed, with sound dentin surrounding the exposure site indicating “mechanical exposure” and thus healthy pulp and carious dentin surrounding the exposure site indicating “carious exposure” and thus pulpal involvement, including considerable inflammation.

Some studies state that pulp that is mechanically exposed has the potential to repair itself, regardless of the diameter of the perforation [14, 15] whereas other studies assert that extensive exposure and tissue destruction may limit healing capacity of pulp [4, 5, 16]. Moreover, in cases of carious exposure, the risk of bacterial contamination increases, which can exacerbate pulp inflammatory status and complicate healing [2, 6].

According to Raslan and Wetzel [17], the etiology of exposure determines pulpal response, making it crucial to distinguish between mechanical and carious exposure. There is limited data about comparison of success of carious and mechanical exposure types [4, 5, 7]. It was claimed that the low success rate of teeth with carious exposure larger than pinpoint size is related to the size and type of exposure, with a large area of exposure surrounded by carious dentin implying a greater risk of bacterial contamination and expansion of inflammation deeper into the pulp [4, 5]. It was also concluded that the low success rates of treatment was also related with the calcium hydroxide (CaOH_2_) used for pulpotomy material [4]. According to Yıldırım et al. [16], under appropriate conditions, spontaneous pulpal healing is possible and this process also depends upon treatment material. In line with these earlier studies, a recent study by Özdemir et al. [7] reported that the outcome of vital pulp therapy is affected not only by exposure type, but also by the choice of pulpotomy agent, which can have an important effect on pulp inflammatory status.

A number of studies have pointed out that a material that is biocompatible has a stable physical structure and possesses the chemical and mechanical properties needed to provide good sealing ability can help prevent the bacterial contamination that causes inflammation and threatens pulp healing capacity, making it possible for pulp to heal and repair itself [2, 17, 18].

In recent years, Mineral Trioxide Aggregate (MTA) has been used for vital pulp therapies [19–28]. MTA is composed mainly of calcium and silica and releases calcium and hydroxide ions when applied to vital tissue. The material has been preferred for vital pulpotomy due to its biocompatibility, antimicrobial activity and superior sealing capacity [19, 20].

The purpose of this study was to evaluate the effect of exposure type (carious or mechanical) on the clinical and radiographical success of MTA pulpotomy in primary molars. The null hypothesis was that the success rate would not vary according to exposure type.

## 2. Materials and Methods

This study was conducted at the Department of Pediatric Dentistry. The study protocol (no. 46/1342) was approved by the faculty's Human Research Ethics Committee. Subjects were selected from among healthy and cooperative children aged 6–9 years requiring pulpotomy treatment for 1 or more primary molars. The study purpose and clinical procedures were explained to the parents, who gave their written informed consent prior to the start of treatment.

Inclusion criteria were as follows: presence of a deep caries lesion (ICDAS 6; extensive cavity with visible dentin); no spontaneous pain; no tenderness to percussion or palpation; no history of swelling or sinus tracts; no radiographic signs of internal/external root resorption, widened periodontal ligament space, or furcal/periapical radiolucency; possibility of restoration with a stainless steel crown (SSC).

**Figure 1:**
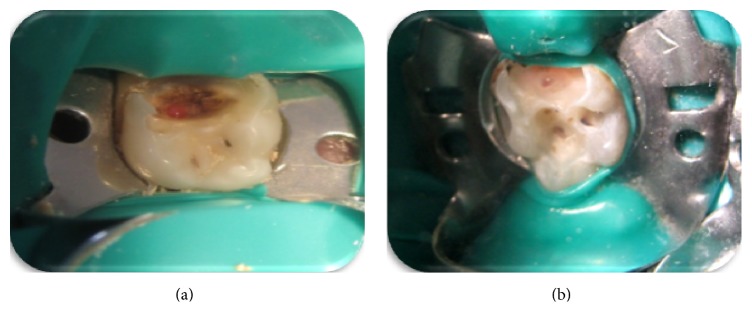
Intraoral views of carious and mechanical exposures. (a) Pulpal exposure surrounded by carious dentin, carious exposure type; (b) pulpal exposure surrounded by sound dentin, mechanical exposure type.

Local anesthesia (Ultracain D-S, Aventis Pharma, İstanbul, Turkey) was administered, and teeth were isolated with a rubber dam. Carious dentin was removed from the pulpal floor, working from the periphery towards the center. At the end of the caries removal process, when pulp was perforated, teeth were grouped as either cariously exposed (Experimental group, *n* = 24), that is, larger than pinpoint pulpal exposure surrounded by carious dentin (Figure 1(a)), or mechanically exposed (control group, *n* = 26), that is, larger than pinpoint pulpal exposure surrounded by sound dentin (Figure 1(b)). Teeth with pulpal exposure smaller than pinpoint, teeth with purulent/viscous, dark-colored exudate detected at the exposure site, and necrotic teeth were excluded from the study.

Pulpotomy treatment in both groups was performed by the same investigator (BNÇ) and following the same procedures. The roof of the pulp chamber was removed, and coronal pulp tissue was removed using a slow-speed instrument and continuous water spray. Following the amputation of the coronal pulp, the cavity was irrigated with saline. Initial hemorrhaging was controlled by placing sterile cotton pellets moisturized with saline over the radicular pulp stump using slight pressure and waiting 5 minutes for hemostasis. MTA paste (ProRoot MTA, Dentsply) was prepared according to the manufacturer's instructions, and the material was used to cover the pulp stumps after the bleeding had stopped. A moistened cotton pellet was placed over the MTA paste to allow the material to set, and the teeth were temporarily restored using reinforced ZOE (ZOE; IRM, Dentsply, York, Pa., USA). After 24 hours, teeth were permanently restored with SSCs.

Subjects were recalled at 3, 6, 12, and 18 months after treatment. Clinical and radiographic examinations were performed at each follow-up visit by 2 examiners blinded to the study groups. An absence of spontaneous pain, pathologic mobility, tenderness to percussion, swelling, fistula, and gingival inflammation was considered as clinical success, whereas absence of internal/external root resorption and periapical/furcal radiolucency was considered as radiographic success. Pulp canal obliteration was not considered as failure.

Clinical and radiographic failures occurring during the follow-up period were treated by pulpectomy or extraction, and space maintainers were applied as necessary. Teeth deemed clinically and radiographically successful were extracted if 2/3 of root growth of the underlying germ was complete.

Data were analyzed using Fisher's exact, Mann–Whitney *U*, and Pearson's chi-square tests with Bonferroni correction.

## 3. Results

**Figure 2:**
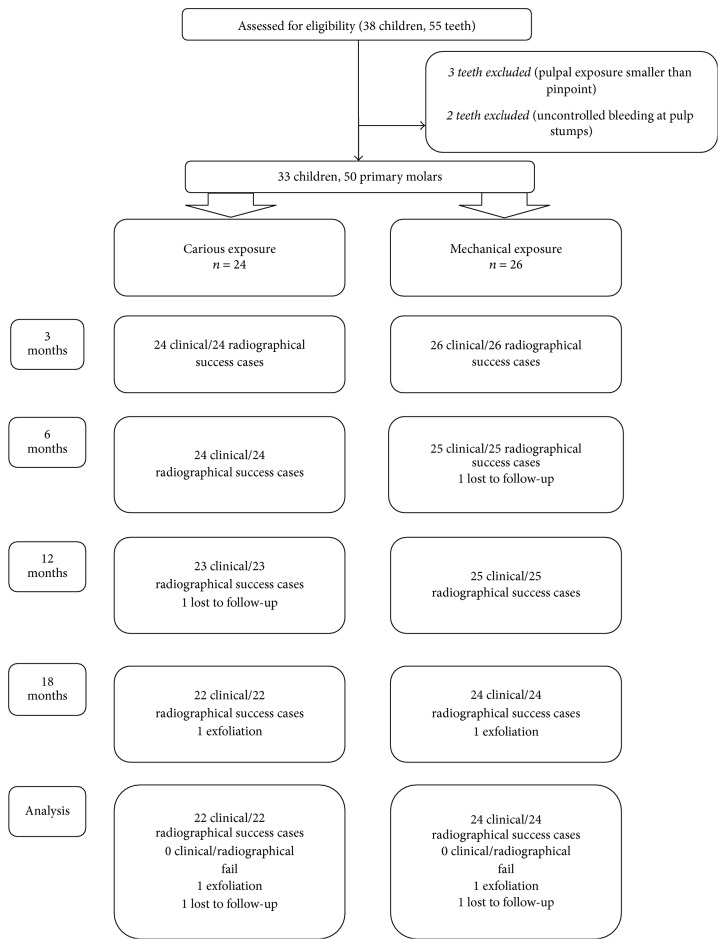
18-month follow-up diagram for carious and mechanical exposure groups.

A total of 50 mandibular primary molar teeth (19 first molar, 31 second molar) diagnosed with deep dentin caries and requiring vital pulpotomy in 33 children (18 girls, 15 boys) aged 6–9 years were included in the study. In terms of exposure etiology, 24 teeth were considered to be cariously exposed and 26 to be mechanically exposed (Table 1). The distribution of teeth according to patient age, gender, and tooth type is given in Table 1. Mann–Whitney *U* and Pearson's Chi-square tests showed homogeneity of age, gender, and tooth type. The diagram in Figure 2 shows the flow of patients and clinical/radiographical success up to the 18-month follow-up visit.

According to clinical and radiographical criteria, success rates were 100% for both groups, so there was no statistically significant difference between the groups at any of the follow-up periods (*p* = 1.000, Table 2). Although not regarded as a failure, pulp canal obliteration was observed in 2 molar teeth in the carious exposure group at 6 months, but radiograph examination showed the teeth had stabilized at 18 months.

## 4. Discussion

While it has been accepted that pulpotomy assessment should be performed differently for cases of mechanical and carious exposure, just how these two conditions should be distinguished has not yet to be clearly defined. Many studies in the literature fail to provide sufficient explanations regarding exposure. Systematic reviews and many studies uncovered various expressions that generalized inclusion criteria as “carious pulp exposures” [21–24, 29]. However, in all of these studies, caries were reportedly removed completely prior to opening of the pulp chamber, which would mean these teeth should be included in Starkey's definition of “mechanical exposure” rather than “carious exposure.” In a small number of other studies in which exposure characteristics were described in detail, carious exposure was found to reduce the success rate of vital pulpotomy treatment [4, 7]. In addition to the nature of exposure, the material used for vital pulpotomy has been shown to have a significant effect on treatment prognosis [7, 13–15].

In light of the above, this study used MTA as a pulpotomy material with the assumption that its well-known anti-inflammatory and sealing properties would be especially useful in cases of carious exposure, where bacterial contamination and thus inflammatory response are severe. In distinguishing between carious and mechanical exposure, this study used Starkey's definitions [13], in which “mechanical exposure” is identified by sound dentin surrounding the exposure site and “carious exposure” by carious dentin surrounding the exposure site.

The literature on pulpotomy offers a conflicting assessment with regard to hemostasis. While the majority of clinical studies consider hemostasis at the pulp stump to be a required criteria for pulpotomy [21, 22, 24–29] a few studies use hemostasis at the exposure site [4–7, 30, 31]. In this study, hemostasis at the pulp stump was required as an inclusion criteria. Bleeding at the exposure site was also assessed, and the presence of purulent or dark bleeding, exudate, or necrosis was used as an exclusion criteria.

Clinical and radiological success rates for MTA pulpotomies have been reported to range from 66.6% to 100% [4, 7, 23, 25, 29]. However, in these studies, inclusion criteria did not provide specific information regarding type of exposure as mentioned above, stating only that exposures after complete removal of decay were included. Thus, it is possible that the reported 66.6%–100% success rates were for teeth with mechanical exposure, which would be in line with the rate found for mechanical exposure (100%) in our study.

At the end of an 18-month follow-up period, our study found 100% success rates for both the mechanical and carious exposure groups. Özdemir et al. [7], which is the only previous study in the literature on MTA pulpotomies to use the same exposure definition as the one used in this study, also reported a 100% success rate for MTA pulpotomies of teeth with carious exposure. Moreover, as in our study, their study found no significant difference in success rates of MTA pulpotomies between cariously (100%) and mechanically (80%) exposed teeth at the end of 18 months. However, the authors did report a significant difference in success rates between cariously and mechanically exposed teeth when calcium hydroxide (CH) was used as a pulpotomy agent (carious: 12.5%; mechanical: 66.7%) as well as a significant difference in CH and MTA pulpotomy success rates for cariously exposed teeth. These results are remarkable in revealing the importance of pulpotomy material, with the superiority of MTA clearly evident. Sönmez and Durutürk [4] reported that the success rate of teeth with larger than pinpoint carious exposure (65.5%) was significantly lower than that of teeth with mechanical exposure (88.5%), and they concluded that the material, CH, was responsible for pulpotomy failure. Other studies have shown clinical and radiographical success rates to be significantly higher with MTA when compared to CH [4, 27, 32–34]. These results have been attributed to the chemical and mechanical stability and superior sealing ability of MTA. Additionally based on the assumption that the inflammatory process is regulated by cytokines [35], the high success rates associated with MTA may be related to its effect on cytokine expression, especially given that the level of inflammation is known to be greater with carious exposure [9, 36–38]. However, MTA is expensive for routine use. Because of the potential complications of other pulpotomy materials, such as internal/external root resorption and periapical/furcal radiolucency and the need for extra procedures such as antiseptic/hemostatic agents prior to the application of material [20, 26], the MTA pulpotomy seems to be the most appropriate pulpotomy material with high success rates.

However it is likely that the success of the MTA group in the present study can be attributed to the material's sealing ability, the role of stainless steel crowns as a coronal restoration was well-known [5, 39], and the results could be related with using the crowns for coronal restoration.

Previous studies have reported a wide variation (0%–58%) in rates of pulp canal obliteration [4, 26, 29]. In our study, obliteration was observed in 2 teeth only, both in the same patient in the carious exposure group (8.3%). Moreover, the obliteration observed at the 6th month of follow-up had stabilized by the 18th month of follow-up; this finding suggests that long term clinical and radiological follow-up of obliteration is required.

In this study, follow-ups were for 18 months and it was thought that long term clinical and histological studies and larger study groups are required.

## 5. Conclusion

The high MTA pulpotomy success rate (100%) was an expected outcome for the mechanical exposure group. Furthermore, the high success rate (100%) obtained for the carious exposure group indicates that MTA can be used in cases where bacterial contamination and related inflammatory response may be more widespread and severe.

Earlier studies, which identified and distinguished the type of pulp exposures, asserted that there should be operative criteria for diagnosis of pulpal status and choice of pulpotomy material could be done by this diagnosis. In addition, according to present study, regardless of the exposure type, long term clinical and radiological success could be expected in the cases of MTA. However, there is a need for additional studies about recent biocompatible materials used for vital pulpotomy in primary teeth and assessment should be done for long term clinical and radiographic success depending on the exposure type.

## Figures and Tables

**Table 1:** Demographic and clinical features of groups.

Variable	Experimental group (carious exposure) (*n* = 24)	Control group (mechanical exposure) (*n* = 26)	*p* value
Age (year)	6–8 (median: 7)	6–9 (median: 7)	0.137^†^
Gender			0.093^‡^
Boy	12 (50.0%)	7 (26.9%)	
Girl	12 (50.0%)	19 (73.1%)	
Tooth type			0.069^‡^
Primary 1st molar	6 (25.0%)	13 (50.0%)	
Primary 2nd molar	18 (75.0%)	13 (50.0%)	

^†^Mann–Whitney *U* test; ^‡^Pearson's chi-square test.

**Table 2:** Success rates of groups, by follow-up period.

Follow-up period	Experimental group (carious exposure) (success/total)	Control group (mechanical exposure) (success/total)	*p*value^†^
3 months	24/24 (100.0%)	26/26 (100.0%)	—
6 months	24/24 (100.0%)	25/25 (100.0%)	1.000
12 months	23/23 (100.0%)	25/25 (100.0%)	1.000
18 months	22/22 (100.0%)	24/24 (100.0%)	1.000

^†^According to Fisher's exact test and Bonferroni correction, values of *p* < 0.0083 were considered statistically significant.
